# Assessment of Human Exposure to Five *Alternaria* Mycotoxins in China by Biomonitoring Approach

**DOI:** 10.3390/toxins13110762

**Published:** 2021-10-28

**Authors:** Kai Fan, Wenbo Guo, Qingwen Huang, Jiajia Meng, Qi Yao, Dongxia Nie, Zheng Han, Zhihui Zhao

**Affiliations:** 1 Key Laboratory of Protected Horticultural Technology, Institute for Agro-Food Standards and Testing Technology, Academy of Agricultural Sciences, Shanghai 201403, China; fankai@saas.sh.cn (K.F.); guo1103bo@126.com (W.G.); huangqingwen@saas.sh.cn (Q.H.); mengjiajia@saas.sh.cn (J.M.); niedongxia@saas.sh.cn (D.N.); hanzheng@saas.sh.cn (Z.H.); 2 Department of Pathology and Pathophysiology, School of Medicine and Life Sciences, Nanjing University of Traditional Chinese Medicine, Nanjing 210023, China; qiqiyao@126.com

**Keywords:** *Alternaria* mycotoxin, human urine, UPLC-MS/MS, risk assessment

## Abstract

This biomonitoring study was conducted to investigate the concentration levels of five *Alternaria* mycotoxins in urine samples from 269 healthy volunteers living in the Yangtze River Delta, China. Alternariol (AOH), alternariol monomethyl ether (AME), tenuazonic acid (TeA) and tentoxin (TEN) were detected in 38.3%, 48.7%, 63.9% and 23.4% of urine samples with the concentrations ranging from 0.057 to 45.8 ng/mL, 0.020 to 0.802 ng/mL, 0.050 to 80.6 ng/mL and 0.021 to 0.939 ng/mL, respectively. Altenuene (ALT) was not detected in any urine sample. Based on the urinary concentrations, the probable daily intake (PDI) values of *Alternaria* mycotoxins were calculated, and 100%, 99.2–100%, 0.372% and 1.12% of participants exceeded the threshold of toxicological concern (TTC) values for AOH, AME, TeA and TEN, respectively. This study revealed high potential health risks related to the contaminations of major *Alternaria* mycotoxins in China and highlighted the necessity for more toxicological studies to provide better basis for further comprehensive risk assessments.

## 1. Introduction

*Alternaria* mycotoxins are secondary metabolites produced by *Alternaria* species, which have been identified as ubiquitously pathogenic genus causing considerable economic losses in worldwide agriculture [1]. Among more than 70 *Alternaria* toxins characterized, alternariol (AOH), alternariol monomethyl ether (AME), altenuene (ALT), tenuazonic acid (TeA), and tentoxin (TEN) are the most important members (Figure S1) [2,3]. The European Food Safety Authority (EFSA) has evaluated *Alternaria* toxins as potentially causing risks to human health and established the threshold of toxicological concern (TTC) values as 2.5 ng/kg bw/day for AOH and AME, and 1500 ng/kg bw/day for TeA and TEN, respectively [4,5].

Due to the strong environmental adaptability, especially growth and production of toxic secondary metabolites at low temperatures, *Alternaria* species may contaminate all stages of the food chain, and *Alternaria* mycotoxins have been frequently detected in numerous food items, including cereals, fruits, dried fruits, vegetables, juices and wine [3,6,7,8,9]. As a consequence, through environments and ingestions of contaminated foods, humans are easily exposed to *Alternaria* mycotoxins. The risks related to the mycotoxins exposure can be estimated based on occurrence data in foods combined with consumption data (external exposure), or, alternatively, via human mycotoxin biomarkers in biological samples (e.g., blood, urine, or breast milk) (biomonitoring) [10,11]. Considering the heterogeneous distribution of mycotoxins in food, the probable insufficient representation of consumption data, and the existence of multiple contamination sources, biomonitoring is recognized as a more effective and valuable approach [12,13,14], and has been successfully conducted to evaluate human exposure to different mycotoxins in Africa, Europe, Asia, and America [15,16,17,18,19]. With regard to *Alternaria* toxins, despite several external exposure assessments [3,5,20,21,22,23], there were only limited reports on the biomonitoring approach. Asam et al., [24] and Hövelmann et al., [25] reported the detection of TeA in German individuals, while AOH was identified in urine samples from Portugal [26]. In China, such biomonitoring data are scarce so far, except for a recent methodological study, in which, an ultrahigh-performance-liquid chromatography tandem mass spectrometry (UPLC-MS/MS) method was developed and preliminarily applied, indicating the presence of *Alternaria* mycotoxins in urine samples [27]. However, the dietary intakes and health risks related to the major *Alternaria* toxins are still in need of investigation.

Therefore, the purpose of the current research was to investigate the occurrence of five important *Alternaria* mycotoxins (AOH, AME, ALT, TeA and TEN) in 269 first morning urine samples from healthy volunteers living in the Yangtze River Delta, China. Therefore, for the first time, the probable daily intake (PDI) values for multiple *Alternaria* mycotoxins were preliminarily estimated and compared with the respective TTC values to characterize the associated health risks.

## 2. Results and Discussion

### 2.1. Method Validation

The performance parameters of the analytical method for the determination of five *Alternaria* mycotoxins in urine were summered in Table 1. The signal suppression/enhancements (SSEs) for AOH, AME and TeA were 54.4%, 70.0% and 58.7%, respectively, verifying significant matrix effects. Thus, matrix-matched calibration curves were established as an alternative accurate quantitation strategy for the lack of the isotope-labelling standards [28]. As urine composition may vary among individuals, it should bear in mind that the uncertainties related to the calculations of the concentrations of *Alternaria* mycotoxins might be relatively higher. Good linear responses for all analytes have been obtained with the coefficients of determination (R^2^) between 0.994 and 0.999 (Table S1). The limit of detection (LOD) and limit of quantification (LOQ) values for individual *Alternaria* mycotoxins ranged from 0.02 to 0.2 ng/mL and 0.05 to 0.5 ng/mL, respectively. The recovery values for *Alternaria* mycotoxins ranged from 75% to 104%, and the maximum intra-day precision and inter-day precision were 9.9% and 11.8%, respectively. Thus, the analytical method was considered as adequate for the analysis of the *Alternaria* mycotoxins in urine.

### 2.2. Levels of Alternaria Mycotoxins in Urine Samples

In total, the analysis of first-morning urine samples from 269 participants revealed the presence of four *Alternaria* mycotoxins (AOH, AME, TeA and TEN) in 87.0% of the samples (Table 2). ALT was not detected in any urine sample. TeA was the most frequent *Alternaria* mycotoxin found in urine, with 63.9% of positive samples at levels ranging from 0.050 to 80.6 ng/mL (0.019 to 63.8 ng/mg creatinine (Cr)), with a mean value of 2.08 ± 7.52 ng/mL (1.82 ± 6.29 ng/mg Cr). AME was the second most frequently detected *Alternaria* mycotoxin (48.7%) at concentrations ranging from 0.020 to 0.802 ng/mL (0.010 to 2.12 ng/mg Cr). AOH and TEN were detected in 38.3% and 23.4% of samples, with the mean values of 1.49 ± 5.05 ng/mL (1.59 ± 5.50 ng/mg Cr) and 0.041 ± 0.105 ng/mL (0.056 ± 0.231 ng/mg Cr), respectively.

The urinary AOH levels in the present work were higher than the results reported in Portugal (frequency 13%, range 0.4–9.91 ng/mL) [26], in Belgium, the Czech Republic, France, the Netherlands and Norway (frequency 7.4%, range 0.025–1.57 ng/mL) [17] and in Nigeria (frequency 6.7%, mean 0.06 ng/mL) [29]; the urinary AME levels were lower than that found in Belgium, the Czech Republic, France, the Netherlands and Norway (frequency 7.4%, range 0.134–22.4 ng/mL) [17]; the urinary TeA levels were lower than the values determined in volunteers from Germany (frequency 97.9–100%, mean 6.58–6.8 ng/mL) [24,25], but higher than those from Belgium, the Czech Republic, France, the Netherlands and Norway (frequency 20.7%, range 0.154–28.5 ng/mL) [17]. The relatively higher concentrations of the major *Alternaria* toxins in the present work compared to the other reports might be due to the subtropical monsoon climate of warm and humid in the Yangtze River Delta, which are more favorable for mycotoxigenic fungi growth and subsequent mycotoxin production [30]. Thus, concerns for *Alternaria* mycotoxins exposure should be raised, according to the present biomonitoring data.

### 2.3. Demographic Variables and Urinary Alternaria Mycotoxin Levels

Distributions of exposure levels for most *Alternaria* mycotoxins were found by Kruskal-Wallis test to be significantly different between regions (Table 2). The detection frequencies and concentrations of AOH and TEN in Hangzhou were apparently higher than that of the other two regions, whereas Shanghai showed higher levels of TeA than Nanjing and Hangzhou. The significant difference may be due to a wide range of factors. Climatic characteristics, environmental and storage conditions could influence the fungi growth and mycotoxin production on food products. Different sampling periods and inter-individual variabilities in absorption, metabolism and excretion might affect the urinary *Alternaria* mycotoxin levels. Moreover, as shown in Table S1, the participants from three cities have different dietary habits. However, no statistically significant correlation between urinary *Alternaria* mycotoxin concentrations and food consumption (*p* < 0.05) was found with various food types (Table S2), which might be caused by the heterogeneous distributions of mycotoxins in foodstuffs, various routes of mycotoxin exposures, and the inaccurate dietary intakes in questionnaires [31]. Thus, further research is needed to determine if specific regional foods are vectors for *Alternaria* mycotoxin exposure.

**Figure 1:**
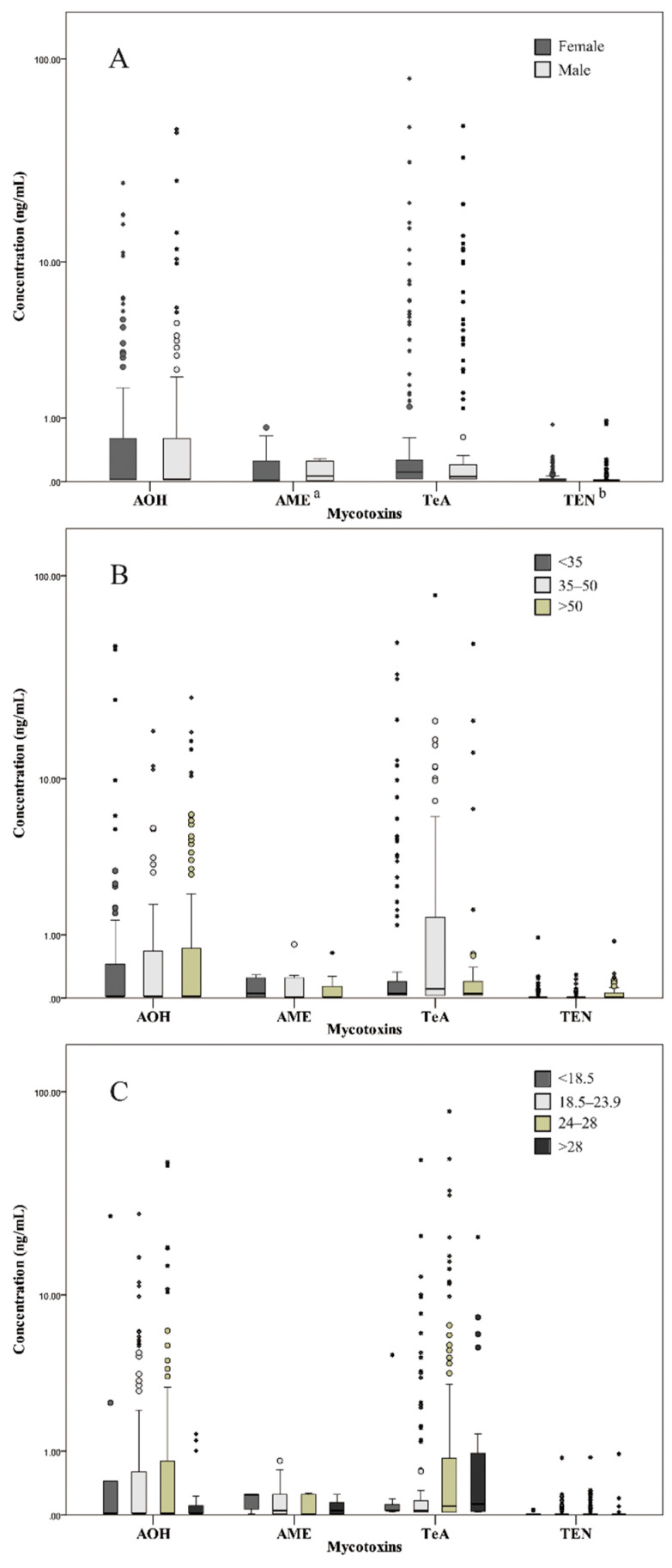
Comparison of *Alternaria* mycotoxin concentrations detected in urine samples, according to (**A**) gender, (**B**) age and (**C**) BMI. The middle lines in the boxes indicate the median; upper and lower box edges indicate the values of the 25th and 75th percentiles. The circles and asterisks correspond to outliers and extremes, respectively. ^a^: *p*-value < 0.01; ^b^: 0.01 < *p*-value < 0.05 by the Mann–Whitney U test. AOH = alternariol; AME = alternariol monomethyl ether; TeA = tenuazonic acid; TEN = tentoxin.

The comparison of urinary *Alternaria* mycotoxin concentrations among different demographic groups (gender, age and body mass index (BMI)) was depicted in Figure 1. Females presented significantly lower urinary AME concentrations than males (*p* < 0.01), while the urinary TEN concentrations in females were higher than those in males (*p* < 0.05). With regard to age and BMI, no significant difference in all *Alternaria* mycotoxin concentrations was found, which was in agreement with the monitoring results of urinary *Alternaria* mycotoxins (AOH, AME and TeA) conducted in European countries [17]. Nevertheless, a number of previous studies described significant demography-related differences in urinary mycotoxin concentrations [31,32,33,34]. Besides the mutability of mycotoxin levels in food, these inconclusive findings might also be attributed to dietary characteristics, body fitness, toxicokinetics and toxicodynamics of the target population. Considering the small number of samples in the present study, further research should be conducted to verify the relationships between urinary *Alternaria* mycotoxin concentrations and demographic factors.

### 2.4. Probable Daily Intake and Risk Characterization

As a kind of “emerging mycotoxins”, there are limited toxicological data on *Alternaria* mycotoxins, and the health-based guidance values (HBGV) were absent yet. Thus, EFSA chose to apply the TTC approach for an initial evaluation for dietary exposure of humans to *Alternaria* mycotoxins [4,35]. In the present study, the PDI values of *Alternaria* mycotoxins were estimated based on the urinary concentrations and compared with respective TTC values to evaluate the health risks. However, it is noticeable a lack of information on toxicokinetics of *Alternaria* mycotoxins. Until now, data on excretion rate derived from human intervention study is only available for TeA. Two volunteers ingested 30 μg TeA by the consumption of naturally contaminated food, and the urinary excretion were determined as 87–93% (mean 89%) after 24 h [24]. For other *Alternaria* mycotoxins, available data were derived from recent studies performed in rats [36,37]. As a consequence, the risk assessment for *Alternaria* mycotoxins in the present study might be considered a rough estimate.

AOH and AME were assigned to the class of potential DNA-reactive substances with a TTC value of 2.5 ng/kg bw/day by EFSA. In the present work, the PDI values for AOH and AME were calculated to be in the range of 3.28–33,700 ng/kg bw/day and 2.33–873 ng/kg bw/day, respectively (Table 3). Similarly, Martins et al. [26] reported the median and maximum values of estimated PDI for AOH in Portugal were 74 and 2451 ng/kg bw/day. These results were in line with the assessment performed by EFSA, which confirmed that the TTC values for AOH and AME were frequently exceeded, indicating that additional toxicity data and further research are required to assess the potential health risks [5,38]. However, it should be noted that the related health risks might be overestimated in the present study for two reasons. First, the PDI values for AOH and AME calculated based on respective LOD/2 values were close to or even higher than the PDI values. Therefore, analytical methods with higher sensitivity were required to more accurately estimate the dietary intake of *Alternaria* mycotoxins. Secondly, the excretion rates derived from rats are low (<9%) and a possible increase of this percentage for humans will decrease the PDIs. Therefore, human excretion rates, based on the excreted amount in 24 h and the calculated dietary intake per day, are required.

Regarding TeA and TEN, the PDI values estimated were in the range of 0.468–1940 ng/kg bw/day and 15.2–2760 ng/kg bw/day, respectively. Only 0.372% and 1.12% of the population exhibited PDI exceeding the assumed TDI value of 1500 ng/kg bw/day, respectively. Similar results were reported in Germany where the PDI values for TeA were 24-1528 ng/kg bw/day (mean 208 ng/kg bw/day) and only for one individual the PDI exceeded the TTC value [25]. These results suggest a low risk to the investigated populations in terms of exposure to TeA and TEN.

## 3. Conclusions

In conclusion, a biomonitoring study was performed to evaluate the health risks related to the contamination of major *Alternaria* mycotoxins in the Yangtze River Delta, China, for the first time. Results indicated a frequent exposure of the investigated Chinese population to four *Alternaria* mycotoxins (AOH, AME, TeA and TEN). The calculated PDI values of selected *Alternaria* mycotoxins were even greater than the TTC values and might represent a potential health concern. Nevertheless, these results should be interpreted carefully due to the uncertainties associated with exposure assessments, especially the excretion rates that were used were derived from only two human individuals (TeA) or rats (AOH, AME and TEN). Another limitation of this study was the collection of first morning urine samples instead of 24 h urine sample, which should be more appropriate and accurate for exposure assessment. However, the collection of complete 24 h urine sample in large population was not convenient, time-consuming, and frequently improper or incomplete [12,39]. As an alternative, first morning urine sample was utilized since it could also provide useful information regarding exposure at the population level as indicated by the previous studies [40,41,42,43]. Moreover, considering the limited number of samples and the lack of *Alternaria* mycotoxins concentrations of adolescents and children, the present work could not represent the entire population in the Yangtze River Delta, China. Therefore, the present work was considered as a pilot survey. Further studies with a larger sample size should be conducted to provide better insights regarding the exposure to *Alternaria* mycotoxins among the general Chinese population.

## 4. Materials and Methods

### 4.1. Reagents and Chemicals

Methanol and acetonitrile were of HPLC grade (Merck Chemicals, Darmstadt, Germany). Ethyl acetate, sodium acetate and ammonium acetate were obtained from Sigma Aldrich (St. Louis, MO, USA). Water was purified on a Milli-Q Plus apparatus (Millipore, Billerica, MA, USA) prior to use. Enzyme β-glucuronidase/arylsulfatase (β-Gluc/ArylS) from *Helix pomatia* was purchased from Roche (Mannheim, Germany). The standards of AOH, AME, ALT, TeA, TEN were purchased from Romer Labs (Union, MO, USA).

### 4.2. Study Populations and Sample Collection

Urine samples were obtained from 269 healthy volunteers (122 females and 147 males) residing in three important cities (Shanghai, Nanjing or Hangzhou) of the Yangtze River Delta, China, between July and November 2019. All participants provided a written consent in accordance with the Helsinki Declaration on ethical principles for medical research involving human subjects, and the study was approved by the local medical ethics committee. During sample collection, the participants were asked to complete a detailed questionnaire with anthropometric information (age, gender, height, and weight), health, occupation and lifestyle factors. Individuals with previous medical records indicating liver, kidney or other metabolic problems were excluded. A food questionnaire on dietary intake in the previous day was administered, in which, typical foods consumed by Chinese people were clearly listed: wheat, maize, rice, vegetables and fruit, meat, nuts and seeds, milk and dairy products, and beverages. The results for the dietary intake of participants are shown in Table S3. The basic demographic characteristics of the study participants are presented in Table S4. The mean age of all participants was 45.4 ± 17.5 years (range 18 to 76 years), and the average BMI of the population was 23.3 ± 3.2 kg/m^2^ (range 16.6–32.9 kg/m^2^). All participants were healthy with no chronic diseases.

The first morning urine sample (~10 mL) was collected from each participant using 50 mL sterile centrifuge tubes (Corning, NY, USA). All urine samples were transported to the laboratory in chilled conditions after collection and stored in a freezer (−20 °C) until extraction.

### 4.3. Sample Preparation

Enzymatic cleavage and extraction of *Alternaria* mycotoxins in urine were performed according to the previous study [27] with minor modifications. Urine samples were thawed at room temperature for 30 min and vortexed for 2 min. Then, 1 mL of each sample was digested with 10 μL β-Gluc/ArylS and 1 mL 0.2 mol/L sodium acetate buffer (PH 5.2–5.3) in water bath at 37 °C overnight. The digested sample was adjusted to about pH 3 with HCl, and then mixed with 5 mL of ethyl acetate and vortexed for 2 min. After centrifugation at 12,000 rpm for 5 min, 2.5 mL of the upper organic phase was transferred into a 10 mL centrifuge tube (Axygen Scientific Inc., Union City, CA, USA), and dried under a stream of nitrogen at 40 °C. The residues were re-dissolved into 250 μL of methanol/water containing 5 mmol/L ammonium acetate (20/80, *v*/*v*) and passed through PTFE membrane syringe filters (0.22 μm) before UPLC-MS/MS analysis.

### 4.4. UPLC-MS/MS Analysis

UPLC-MS/MS analysis was conducted on a Waters Acquity UPLC system (Waters, Milford, MA, USA) and an AB SCIEX QTRA^®^ 5500 tandem mass spectrometer (AB SCIEX instruments, Foster City, Canada). Separation was achieved on a Waters XBridge^®^ BEH C18 Column (3.0 mm × 100 mm, 2.5 μm) with the mobile phase consisting of methanol (A) and water containing 5 mmol/L ammonium acetate (B). A linear gradient elution program was designed as follows: initial 10% (A), 1 min 10% (A), 5 min 90% (A), 6 min 10% (A), 7 min 10% (A) and hold on for a further 2 min for re-equilibration, yielding a total run time of 9 min. The flow rate was 0.4 mL/min, and the injection volume was 3 μL. The column temperature and sample temperature were maintained at 40 °C and 5 °C, respectively. Alternaria mycotoxins were analyzed by MS/MS with the electrospray ionization source operated in both positive (ESI^+^) and negative (ESI^−^) modes. The parameters were set as follows: ion spray voltage, 5.5 kV (positive ion mode) and 4.5 kV (negative ion mode); source temperature, 500 °C; declustering potential, 96 V; entrance potential, 8 V; exit potential, 12 V; curtain gas, 35 psi; ion source gas 1, 50 psi; ion source gas 2, 50 psi; collision gas, 8 psi. Multiple reaction monitoring (MRM) acquisition mode was applied for the determination of the targeted analytes. The parameters and collision energies of precursor and product ions are listed in Table S5. MRM peak integrations and data analysis were carried out using the MultiQuant algorithm from MultiQuant 3.0.2 (AB SCIEX, Foster City, CA, USA).

### 4.5. Method Validation

The established method was validated by determining the linearity, LOD, LOQ, recovery and precision to ensure its sensitivity, accuracy and repeatability, following Commission Regulation 2006/401/EC [44].

*Alternaria* mycotoxin standards were spiked into methanol/water containing 5 mmol/L ammonium acetate (20/80, *v*/*v*) and into blank urine matrices with a concentration sequence of 0.02, 0.05, 0.1, 0.2, 0.5, 1, 2, 5, 10, 20, 50 and 100 ng/mL. Calibrations curves (1/x weighted) were created for each analyte by plotting the responses versus the concentrations. The SSE, which was calculated by comparing the slope of the matrix-matched calibration curve with that of the standard calibration curve, was used to assess the matrix effects. The sensitivity was assessed by determining LOD and LOQ, which were designed as the concentrations of the analyte in matrix that could provide a signal-to-noise ratio (S/N) of 3/1 for the second intense transition and 10/1 for the most intense transition, respectively. The recovery, intra- and inter-day precision tests were all performed in five replicates using blank urine samples spiked with low, intermediate and high concentration levels (0.05, 0.2 and 2 ng/mL for AME and TEN; 0.1, 0.5 and 5 ng/mL for AOH and TeA; 0.5, 2 and 10 ng/mL for ALT). Recovery was calculated by comparing the measured concentration using the matrix-matched calibration curves with the spiked (theoretical) concentration of each analyte. Recoveries between 70% and 120% were considered acceptable. The relative standard deviations (RSDs) determined in the same day were used for evaluation of the intra-day precision, whereas the values in five consecutive days were used for inter-day precision.

### 4.6. Creatinine Analysis

To account for the variability in urine dilution between individual samples, the urinary concentrations of *Alternaria* mycotoxins were normalized to Cr, which was determined using an enzymatic reaction on a Roche Hitachi 912 Chemistry Analyzer (Roche Hitachi, Basel, Switzerland). According to the proposal of the World Health Organization (WHO) [45], creatinine levels in the range of values (30–300 mg/dL) were considered acceptable for biomonitoring.

### 4.7. Estimated Dietary Exposure of Alternaria Mycotoxins

The PDI of the *Alternaria* mycotoxin was calculated based on the measured urinary concentration according to equation below [25]:

(1) $$\mathrm{PDI}\;(\mathrm{ng}/\mathrm{kg}\;•\;\mathrm{bw}/\mathrm{day})\;=\;\frac{C\;\times \;V\;\times \;100}{W\;\times \;E}$$

*C*: the concentration of *Alternaria* mycotoxin in urine (ng/L), *V*: the mean volume of daily urine excretion (1.5 L/day) [39], *W*: individual body weight (kg), *E*: mean urinary excretion rate of *Alternaria* mycotoxin (%).

### 4.8. Statistical Analysis

All the statistical analyses were performed using IBM SPSS Statistics software version 22 (SPSS Inc., Chicago, IL, USA). For statistical analysis, urinary concentrations between LOD and LOQ were replaced with LOQ/2 and the concentrations below LOD were replaced with LOD/2. Differences of mycotoxin concentrations among subgroups were tested with the non-parametric Mann–Whitney U test or Kruskal–Wallis test, and correlation analysis was conducted using Spearman’s rank correlation coefficient (two-tailed). A *p* value < 0.05 was considered statistically significant.

## Figures and Tables

**Table 1:** Method validations for the five *Alternaria* mycotoxins in urine samples.

Mycotoxins	LOD (ng/mL)	LOQ (ng/mL)	SSE (%)	Spiked Levels (ng/mL)	Recovery (%)	Intra-Day Precision (%)	Inter-Day Precision (%)
AOH	0.04	0.1	54.4	0.1	93	2.4	10.7
				0.5	85	5.8	5.4
				5	91	2.1	5.2
AME	0.02	0.05	70.0	0.05	75	6.7	8.0
				0.2	93	3.4	2.3
				2	92	6.2	1.8
ALT	0.2	0.5	88.7	0.5	104	5.9	10.8
				2	101	3.5	4.3
				10	100	9.9	4.6
TeA	0.05	0.1	58.7	0.1	77	7.6	11.8
				0.5	90	6.7	7.7
				5	97	9.6	11.7
TEN	0.02	0.05	84.1	0.05	90	9.5	8.6
				0.2	90	2.6	3.3
				2	101	2.3	3.3

AOH = alternariol; AME = alternariol monomethyl ether; ALT = altenuene; TeA = tenuazonic acid; TEN = tentoxin; LOD = limit of detection, determined at signal-to-noise ratio (S/N) = 3; LOQ = limit of quantification, determined at S/N = 10; SSE = signal suppression/enhancement, calculated by comparing the slope of the matrix-matched calibration curve with that of the standard calibration curve; recovery, intra- and inter-day precision, performed in five replicates using blank urine samples spiked with low, intermediate and high concentration levels.

**Table 2:** Occurrence of AOH, AME, TeA and TEN in urine samples in the Yangtze River Delta, China.

Mycotoxins	All (*n* = 269)	Shanghai (*n* = 93)	Nanjing (*n* = 86)	Hangzhou (*n* = 90)	*p*-Value ^a^
AOH					
Frequency % (*n*)	38.3 (103)	36.6 (34)	30.2 (26)	47.8 (43)	
Mean ± SD (ng/mL)	1.49 ± 5.05	0.760 ± 2.80	0.235 ± 0.476	3.46 ± 7.90	0.001
Range (ng/mL)	0.057–45.8	0.061–25.0	0.057–3.02	0.088–45.8	
Mean ± SD (ng/mg Cr)	1.59 ± 5.50	0.855 ± 4.46	0.270 ± 0.554	3.61 ± 7.98	0.004
Range (ng/mg Cr)	0.037–43.9	0.084–42.6	0.044–3.13	0.037–43.9	
AME					
Frequency % (*n*)	48.7 (131)	53.8 (50)	51.2 (44)	41.1 (37)	
Mean ± SD (ng/mL)	0.102 ± 0.120	0.113 ± 0.131	0.111 ± 0.113	0.082 ± 0.113	0.140
Range (ng/mL)	0.020–0.802	0.027–0.802	0.020–0.171	0.020–0.640	
Mean ± SD (ng/mg Cr)	0.111 ± 0.188	0.101 ± 0.125	0.115 ± 0.142	0.117 ± 0.267	0.116
Range (ng/mg Cr)	0.010–2.12	0.010–0.406	0.011–0.193	0.010–2.12	
TeA					
Frequency % (*n*)	63.9 (172)	75.3 (70)	74.4 (64)	42.2 (38)	
Mean ± SD (ng/mL)	2.08 ± 7.52	2.94 ± 8.32	1.68 ± 4.49	1.57 ± 8.85	0.000
Range (ng/mL)	0.050–80.55	0.052–47.5	0.05–33.4	0.052–80.6	
Mean ± SD (ng/mg Cr)	1.82 ± 6.29	2.41 ± 7.82	2.00 ± 4.95	1.06 ± 5.60	0.001
Range (ng/mg Cr)	0.019–63.8	0.019–63.8	0.032–29.0	0.093–50.4	
TEN					
Frequency % (*n*)	23.4 (63)	20.4 (19)	11.6 (10)	37.8 (34)	
Mean ± SD (ng/mL)	0.041 ± 0.105	0.026 ± 0.041	0.018 ± 0.031	0.079 ± 0.169	0.000
Range (ng/mL)	0.021–0.939	0.021–0.226	0.026–0.239	0.025–0.939	
Mean ± SD (ng/mg Cr)	0.056 ± 0.231	0.026 ± 0.053	0.020 ± 0.034	0.123 ± 0.387	0.003
Range (ng/mg Cr)	0.016–2.86	0.016–0.417	0.022–0.223	0.026–2.08	

AOH = alternariol; AME = alternariol monomethyl ether; TeA = tenuazonic acid; TEN = tentoxin; Cr = creatinine. ^a^ *p*-values obtained using the Kruskal–Wallis test.

**Table 3:** Estimate of probable daily intakes of *Alternaria* mycotoxins based on urinary concentrations.

Mycotoxins	Excretion Rate		PDI (ng/kg bw/day)					TTC (ng/kg bw/day)	Exceeding TTC (%)
	ER (%)	Reference	Min	Mean	Median	P95	Max		
AOH	8.3	Puntscher et al. [36] ^a^	3.28	419	6.57	1880	11,400	2.5	100
	2.8	Puntscher et al. [37] ^a^	9.74	1240	19.5	5580	33,700		100
AME	6.7	Puntscher et al. [36] ^a^	2.33	36.8	4.76	105	339	2.5	99.2
	2.6	Puntscher et al. [37] ^a^	6.01	94.8	12.3	272	873		100
TeA	89	Asam et al. [24] ^b^	0.468	52.1	1.75	284	1940	1500	0.372
TEN	0.9	Puntscher et al. [37] ^a^	15.2	107	27.8	442	2760	1500	1.12

AOH = alternariol; AME = alternariol monomethyl ether; TeA = tenuazonic acid; TEN = tentoxin; ER = excretion rate; nPDI = probable daily intake; TTC = threshold of toxicological concern. ^a^ Studies performed in rats. ^b^ Study including two volunteers.

## Data Availability

The data presented in this study are available at https://www.mdpi.com/journal/toxins/special_issues/Biomonitoring_Assessment_Mycotoxins, accessed date 25 October 2021.

## Supplementary Materials

The following are available online at https://www.mdpi.com/article/10.3390/toxins13110762/s1, Figure S1: chemical structures of the Alternaria mycotoxins, Table S1: linear range and correlation coefficient (R2) for the five Alternaria mycotoxins in urine samples, Table S2: correlations (r) between urinary Alternaria mycotoxin levels and food consumptions, Table S3: food consumptions of the participants, Table S4: demographic characteristics of the participants, Table S5: MS/MS parameters for five *Alternaria* mycotoxins.
