# Supplementary material for: Assessment of Human Exposure to Five Alternaria Mycotoxins in China by Biomonitoring Approach
Source: Toxins (Basel). 2021 Oct 28;13(11):762. doi: 10.3390/toxins13110762 (PMC8625692; doi:10.3390/toxins13110762)
Supplement: Supplementary file 1 [file toxins-13-00762-s001.zip › toxins-1395720-supplementary.pdf]

toxins

Supplementary Information

Assessment of Human Exposure to Five *Alternaria* Mycotoxins in China by Biomonitoring Approach

Kai Fan [1], Wenbo Guo [1], Qingwen Huang [1], Jiajia Meng [1], Qi Yao [2], Dongxia Nie [1], Zheng Han [1] and Zhihui Zhao [1,*]

[1] Key Laboratory of Protected Horticultural Technology, Institute for Agro-food Standards and Testing Technology, Academy of Agricultural Sciences, Shanghai 201403, China; fankai@saas.sh.cn (K.F.); guo1103bo@126.com (W.G.); huangqingwen@saas.sh.cn (Q.H.); mengjiajia@saas.sh.cn (J.M.); niedongxia@saas.sh.cn (D.N.); hanzheng@saas.sh.cn (Z.H.)
[2] Department of Pathology and Pathophysiology, School of Medicine and Life Sciences, Nanjing University of Traditional Chinese Medicine, Nanjing, 210023 Jiangsu Province, China; qiqiyao@126.com
* Correspondence: zhaozhihui@saas.sh.cn; Tel.: +86-21-62202875

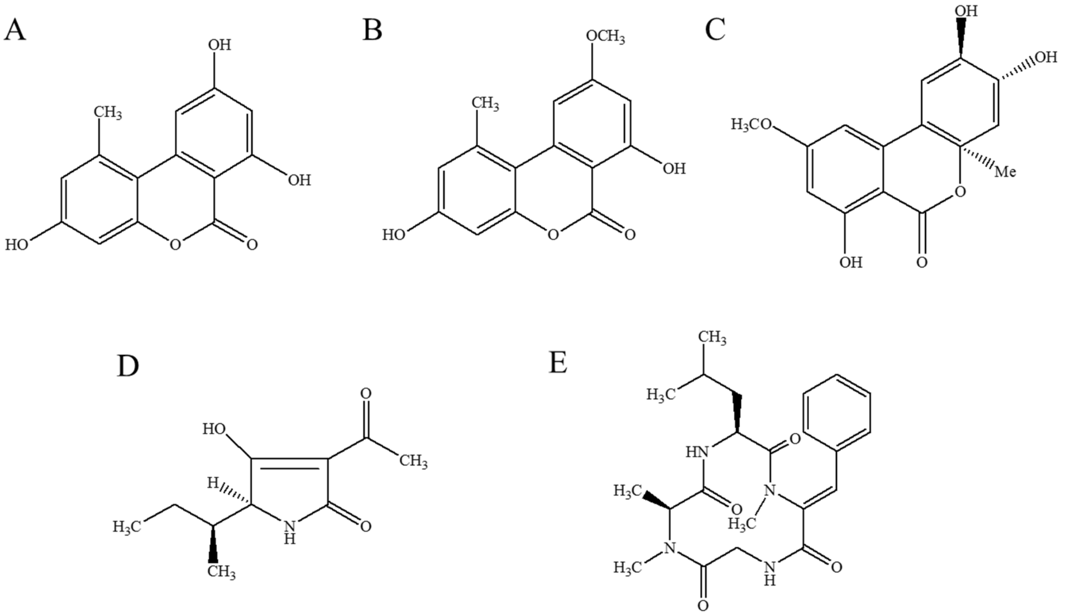

Figure S1. Chemical structures of the *Alternaria* mycotoxins: (A) alternariol (AOH); (B) alternariol monomethyl ether (AME); (C) altenuene (ALT); (D) tenuazonic acid (TeA); (E) tentoxin (TEN).

Table S1. Linear range and correlation coefficient (R^2) for the five *Alternaria* mycotoxins in urine samples.

Mycotoxin	Range (ng/mL)	R^2
AOH	0.1–100	0.997
AME	0.05–50	0.997
ALT	0.5–100	0.998
TeA	0.1–100	0.994
TEN	0.05–50	0.999

Table S2. Correlations (*r*) between urinary *Alternaria* mycotoxin levels and food consumptions.

Foods	AOH		AME		TEA		TEN	
	r	*p*	*r*	*p*	*r*	*p*	*r*	*p*
Rice	0.105	0.087	−0.018	0.764	−0.114	0.062	0.059	0.338
Wheat	−0.092	0.134	0.072	0.240	0.057	0.354	0.026	0.671
Maize	−0.086	0.159	−0.001	0.989	0.005	0.944	−0.067	0.275
Vegetables and fruit	0.067	0.275	−0.042	0.497	0.031	0.617	−0.006	0.919
Meat	−0.012	0.848	0.025	0.685	−0.047	0.442	−0.020	0.748
Nuts and seeds	0.062	0.311	−0.071	0.241	−0.026	0.669	0.048	0.435
Milk and dairy produce	−0.096	0.117	0.003	0.967	0.063	0.304	−0.006	0.922
Beverages	−0.038	0.539	0.044	0.474	−0.023	0.702	0.014	0.822

Table S3. Food consumptions of the participants.

Food Groups	24 h Consumption (g or mL)				*p* [a]
	All	Shanghai	Nanjing	Hangzhou	
Rice	190.5	146.8	191.9	234.4	0.000
Wheat	104.2	104.6	114.2	94.2	0.126
Maize	16.4	5.6	37.8	7.0	0.000
Vegetables and fruit	165.9	182.3	125.3	187.8	0.059
Meat	119.0	110.5	117.7	128.9	0.623
Nuts and seeds	28.4	24.2	27.8	33.3	0.001
Milk and dairy produce	48.5	63.4	29.1	51.7	0.002
Beverages	135.3	121.5	182.3	104.4	0.024

[a] The *p*-value was calculated using the Kruskal-Wallis test.

Table S4. Demographic characteristics of the participants.

Characteristics.	All	Shanghai	Nanjing	Hangzhou
		**Gender (n)**		
Female	122	45	39	38
Male	147	48	47	52
All	269	93	86	90
		**Age (years)**		
Mean ± SD	41.6 ± 14.5	40.4 ± 13.5	37.3 ± 10.4	47.0 ± 17.1
Range	18–76	23–75	18–66	21–76
		**BMI (kg/m^2)**		
Mean ± SD	23.3 ± 3.1	23.0 ± 3.5	23.4 ± 3.0	23.6 ± 2.7
Range	16.6–32.9	16.6–32.9	18.1–30.5	18.2–29.4

Table S5. MS/MS parameters for five *Alternaria* mycotoxins.

Mycotoxins	Precursor ion (*m/z*)	Product ion (*m/z*)	Retention Time (min)	Collision Energy (eV)
AOH	257	215	5.86	−27
		147 [a]		−33
AME	271	256 [a]	6.86	−30
		227		−40
ALT	293	239 [a]	5.42	20
		229		29
TeA	196	139 [a]	3.36	−22
		112		−24
TEN	415	312 [a]	6.17	30
		256		40

[a] Quantifier ion.
